# Divergent endometrial inflammatory cytokine expression at peri-implantation period and after the stimulation by copper intrauterine device

**DOI:** 10.1038/srep15157

**Published:** 2015-10-15

**Authors:** Chia-Hung Chou, Shee-Uan Chen, Chia-Tung Shun, Po-Nien Tsao, Yu-Shih Yang, Jehn-Hsiahn Yang

**Affiliations:** 1 Department of Obstetrics and Gynaecology, National Taiwan University Hospital and National Taiwan University College of Medicine, Taipei, 100, Taiwan; 2 Department and Graduate Institute of Forensic Medicine, National Taiwan University Hospital and National Taiwan University College of Medicine, Taipei, 100, Taiwan; 3 Department of Paediatrics, National Taiwan University Hospital and National Taiwan University College of Medicine, Taipei, 100, Taiwan; 4 Research Centre for Development Biology & Regenerative Medicine, National Taiwan University, Taipei, 106, Taiwan

## Abstract

Endometrial inflammation has contradictory effects. The one occurring at peri-implantation period is favourable for embryo implantation, whereas the other occurring after the stimulation by copper intrauterine device (Cu-IUD) prevents from embryo implantation. In this study, 8 week female ICR mice were used to investigate the endometrial inflammation, in which they were at proestrus stage (Group 1), at peri-implantation period (Group 2), and had a copper wire implanted into right uterine horn (Group 3). Cytokine array revealed that two cytokines were highly expressed in Group 2 and Group 3 as compared with Group 1, and seven cytokines, including tumour necrosis factor α (TNF-α), had selectively strong expression in Group 3. Immunohistochemistry demonstrated prominent TNF-α staining on the endometrium after Cu-IUD stimulation, and *in vitro* culture of human endometrial glandular cells with Cu induced TNF-α secretion. The increased TNF-α concentration enhanced *in vitro* THP-1 cells chemotaxis, and reduced embryo implantation rates. These results suggest that **i**nflammatory cytokine profiles of endometrium are different between those at peri-implantation period and after Cu-IUD stimulation, and TNF-α is the one with selectively strong expression in the latter. It might account for the contradictory biological effects of endometrial inflammation.

Embryo implantation is a critical step in human reproduction. However, the embryo implantation rate remains relatively low even with *in vitro* fertilisation treatment. Endometrial inflammation occurs at the peri-implantation period, in which there are lymphocyte infiltration and increased expression of several inflammatory cytokines including IL-1α, tumour necrosis factor α (TNF-α), matrix metalloproteinase 9 (MMP-9), colony stimulating factor 1 (CSF-1), leukocyte inhibitory factor (LIF), and IL-11^1^. High levels of pro-inflammatory cytokines IL-6, IL-8, and TNF-α have been found to characterise early embryo implantation. They are secreted by either endometrial cells or immune cells that are recruited to the site of embryo implantation^2^,^3^. As in any inflammation, the endometrial inflammation is accompanied by the induction of adhesion molecules that play an important role in establishing an embryo implantation environment^4^.

Copper intrauterine device (Cu-IUD) is one of the most effective forms for birth control and has been used for a couple of decades. Several mechanisms have been proposed to account for its contraceptive action, including the prevention of sperm from reaching fallopian tubes, the hindrance to fertilisation^5^,^6^, and the induction of enhanced inflammatory response with increased numbers of leukocytes^5^,^7^. Cu-IUD has also been found to alter cytokine and integrin expression in the endometrium, and consequently interferes with embryo implantation^8^,^9^.

It appears that the endometrial inflammation has contradictory results. One is favourable for embryo implantation, as is shown at the peri-implantation period, whereas the other prevents from embryo implantation, as is noted in the case of Cu-IUD stimulation. Inflammation is a biological response of vascular tissues to harmful stimuli, which is mediated by a variety of molecules. Accordingly, it would be of value to study the different cytokine profiles of endometrial inflammation between those at peri-implantation period and after Cu-IUD stimulation. Results obtained after our experiments might provide important information with regard to the factors influencing embryo implantation.

## Results

### Animals and Grouping

**Figure 1:**
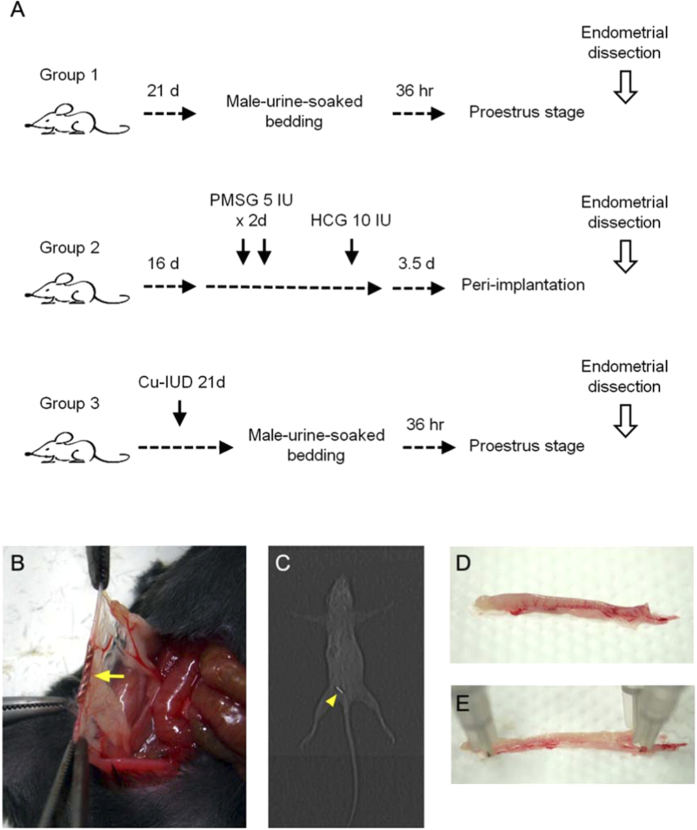
(**A**) Brief protocols of the three groups are demonstrated. (**B**) Midline laparotomy is performed and a copper wire with the length of 5 mm and a diameter of 0.3 mm is implanted into the right uterine horn (arrow) in a 8-week-old ICR female mouse for 21 days. (**C**) The location of copper wire is confirmed with computed tomography (arrowhead). (**D**) The right uterine horn is dissected and cut open (**E**), and the endometrium is obtained by curettage.

Female ICR mice at 8 week old and weighing ~20 g were used as the experimental subjects. They were divided into 3 groups, in which Group 1 mice were the controls at proestrus stage; Group 2 mice were at peri-implantation period; and those in Group 3 had a copper wire implanted into their right uterine horn (Fig. 1A). This study protocol was approved by the Institutional Animal Care and Use Committee (IACUC) at College of Medicine and College of Public Health, National Taiwan University. All experiments were carried out in accordance with the approved guidelines.

For Group 2 mice, intraperitoneal PMSG 5 IU was injected for 2 consecutive days, followed by hCG 10 IU injection, and endometrial tissue was obtained 3.5 days later, roughly at the peri-implantation endometrium. For Group 3 mice, inhalational isoflurane (2-chloro-2-(difluoromethoxy)-1,1,1-trifluoro-ethane) anaesthesia was done, followed by a midline laparotomy. A copper wire obtained from Nova-T^®^ IUD (Bayer Schering Pharma, Turku, Finland) with the length of 5 mm and a diameter of ~0.3 mm was implanted into the right uterine horn for 21 days (Fig. 1B). The location of copper wire was confirmed with computed tomography (Fig. 1C).

Whitten effect^10^ was achieved with male-urine-soaked bedding in Group 1 and Group 3 for estrus cycle synchronisation. Uterine dissection was done 36 hours later, when the endometrium was supposed to be at the proestrus stage^11^.

### Protein preparation

Female mice (n = 5 in each group) were anaesthetised with isoflurane, and the uterus was dissected (Fig. 1D) and cut open (Fig. 1E). The endometrium was obtained by curettage and was ground into fine powder with a pestle and mortar, and then resuspended in 10 mM EDTA, 0.4% 2-mercaptoethanol, 0.9 M sucrose, and 0.1 M Tris-HCl at pH 7.8. The homogenate was centrifuged at 5000 × *g* thereafter for 15 minutes at 4 °C. Finally, the supernatant was kept at −80 °C.

### Expression profiles of endometrial inflammation

**Figure 2:**
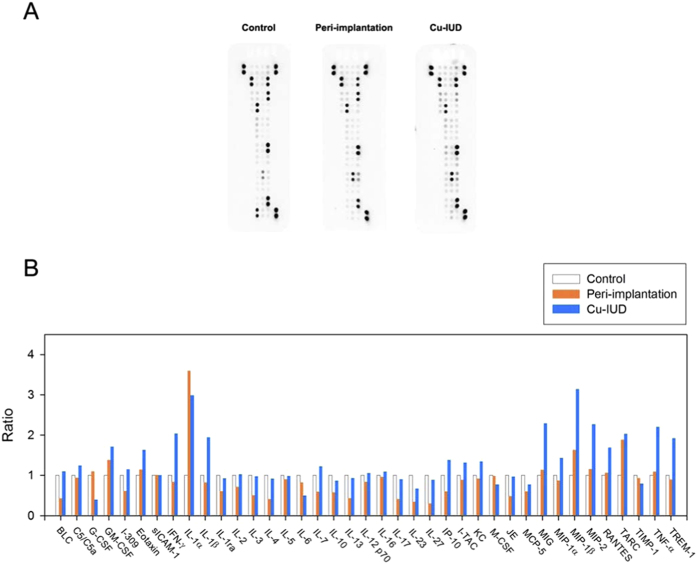
(**A**) Signals of cytokine antibody array are shown. (**B**) Relative expression ratio of Group 2 (at peri-implantation period) and Group 3 (after Cu-IUD stimulation) is calculated using Group 1 (control group) as the benchmark. Using the cutoff value of 1.8, IL-1α and thymus and activation regulated chemokine (TARC) are more strongly expressed both in Group 2 and Group 3 as compared with those in Group 1. By contrast, TNF-α, IFN-γ, IL-1β, monokine induced by gamma interferon (MIG), macrophage inflammatory protein 1β (MIP-1β), MIP-2, and triggering receptor expressed on myeloid cells 1 (TREM-1) are more strongly expressed after the stimulation by Cu-IUD than those in the other two groups.

Mouse cytokine array (Proteome Profiler™, R&D Systems) was used to analyse the protein profiles. Signals of cytokine array are shown in Fig. 2A. Quantification of the expression intensity was done using Group 1 as the benchmark, and the relative expression ratios of Group 2 and Group 3 were calculated. Using the cutoff value of 1.8, it revealed that IL-1α and thymus and activation regulated chemokine (TARC) were more strongly expressed both in Group 2 and Group 3, as compared with those in Group 1. By contrast, TNF-α, interferon γ (IFN-γ), IL-1β, monokine induced by gamma interferon (MIG), macrophage inflammatory protein 1β (MIP-1β), MIP-2, and triggering receptor expressed on myeloid cells 1 (TREM-1) were more strongly expressed after the stimulation by Cu-IUD than those in the other two groups (Fig. 2B).

### Histology of endometrium

**Figure 3:**
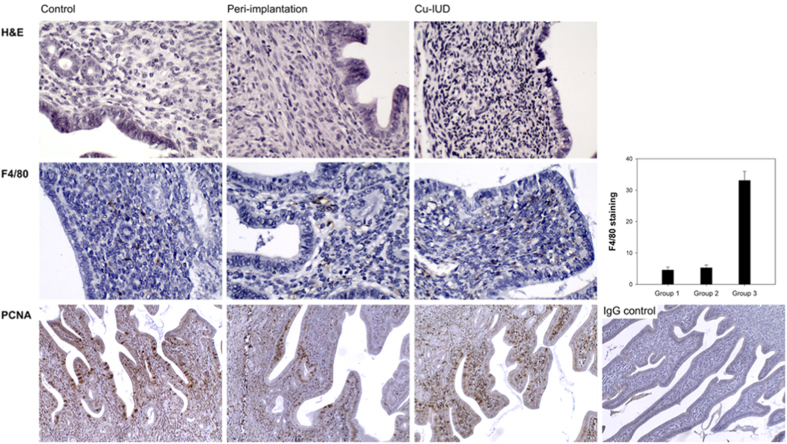
Haematoxylin and eosin (H & E) stain demonstrates subepithelial lymphocytic infiltration in the endometrium at peri-implantation period (Group 2) and after the stimulation by Cu-IUD (Group 3), but not evident in the control group (Group 1). The lymphocytic infiltration is more prominent in Group 3 than that in Group 2. Immunohistochemisty shows more obvious F4/80 (macrophage) staining (n = 10, p < 0.001) in Group 3 than that in Group 1 and Group 2, whereas the proliferating cell nuclear antigen (PCNA) staining is not different among the three groups.

Haematoxylin and eosin (H&E) stain revealed subepithelial lymphocytic infiltration in the endometrium of Group 2 and Group 3, but not evident in Group 1. The lymphocytic infiltration is strinkingly prominent in Group 3 as compared with that in Group 2. Immunohistochemisty identified prominent F4/80 (a well known and widely used marker of murine macrophages) staining in Group 3, but not in Group 1 and Group 2. The mean number of F4/80 immunopositive cells were 33.1 in Group 3 per field with a ×400 magnification, much higher than those in Group 1 and 2 (4.6 and 5.3 respectively, p < 0.001). On the other hand, staining of proliferating cell nuclear antigen (PCNA) was not different among endometria of the three groups (Fig. 3).

### Cu-induced TNF-α production in human endometrial glandular cells (hEGCs)

The contraceptive effect of Cu-IUD is based on the release of cupric ion (Cu^2+^) to endometrial and oviductal fluids^12^. As a result, hEGCs were treated with different concentrations of CuSO_4_ (0, 10, 50, 100 μM) to evaluate the effect of Cu^2+^ on the induction of TNF-α in hEGCs. The cell culture supernatant was collected 24 hours later, and TNF-α concentrations were determined by a mouse TNF-α Quantikine ELISA Kit (MTA00B, R & D Systems).

**Figure 4:**
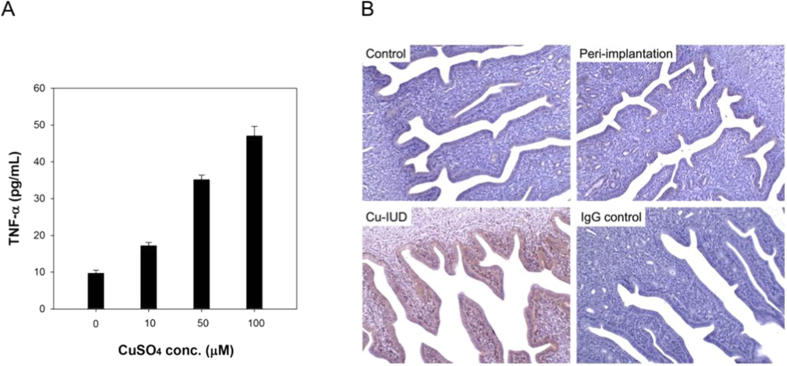
(**A**) *In vitro* treatment of human endometrial glandular cells (hEGCs) with CuSO_4_ achieves a dose-dependent release of TNF-α (n = 3). The higher concentrations of CuSO_4_, the more TNF-α is produced by hEGCs (p < 0.001). (**B**) Immunohistochemisty reveals prominent TNF-α expression in the endometrium after Cu-IUD stimulation (Group 3), but not in the other two groups.

It showed that the higher concentrations of CuSO_4_ treatment, the more TNF-α was produced by hEGCs (p < 0.001 by ANOVA, Fig. 4A). Immunohistochemisty revealed that TNF-α was heavily stained in the mouse endometrium after Cu-IUD stimulation, but not in the control group and at peri-implantation period (Fig. 4B).

### Chemotaxis assay

**Figure 5:**
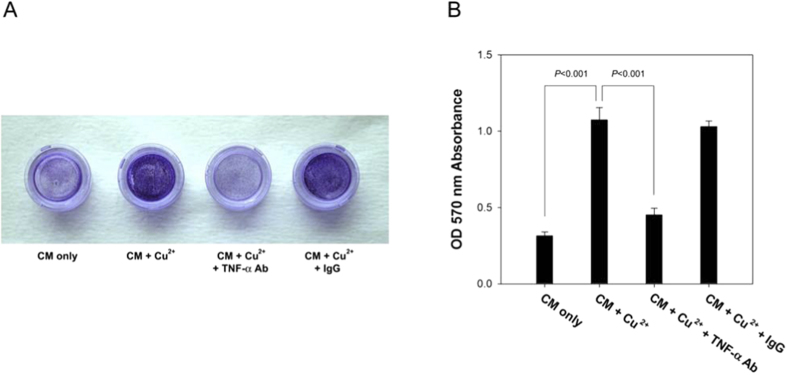
(**A**,**B**) *In vitro* chemotaxis assay (n = 4) shows that conditioned medium (CM) obtained from Cu-treated human endometrial glandular cells (hEGCs) significantly enhances chemotaxis of THP-1 cells, as compared with that obtained from hEGCs without Cu treatment (p < 0.001), and the addition of TNF-α neutralising antibody reduces chemotactic effect (p < 0.001).

Chemotaxis assay was done with 24-well transwell inserts, in which the trans-well migration of human THP-1 cells toward conditioned media was examined. THP-1 is a human monocytic cell line that is widely used to investigate the differentiation of monocytes into macrophages^13^. Blue spots developed in positions at which cell migration had occurred (Fig. 5A). It revealed that the conditioned medium obtained from Cu-treated hEGCs significantly enhanced the chemotaxis of THP-1 compared with that obtained from Cu-free hEGCs (p < 0.001). As the conditioned medium was pretreated with TNF-α neutralising antibody, the chemotaxis of THP-1 cells decreased (p < 0.001, Fig. 5B).

### *In vitro* embryo implantation assay

**Figure 6:**
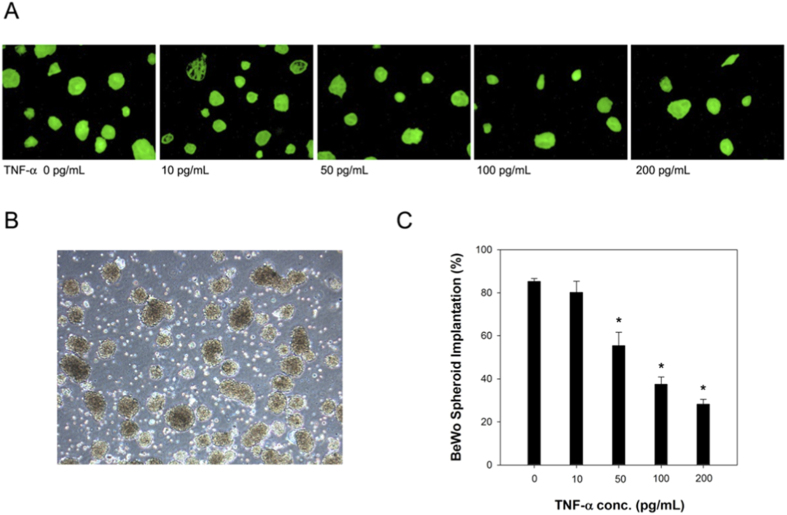
BeWo spheroids formed spontaneously by cell aggregation after 24 hours of culture (**B**). *In vitro* BeWo spheroid- human endometrial glandular cells (hEGCs) implantation assay (n = 3) demonstrates that embryo implantation potentials decrease gradually as hEGCs are treated with higher TNF-α concentrations. When hEGCs are treated with 50 (p = 0.003 and 0.011 respectively), 100 (p < 0.001), and 200 (p < 0.001) pg/mL TNF-α, the embryo implantation rates are much lower than those with 0, and 10 pg/mL TNF-α treatment (**A**,**C**).

*In vitro* BeWo spheroid-hEGCs implantation assay demonstrated that embryo implantation potentials were similar between hEGCs treated with 10 pg/mL TNF-α and those without TNF-α treatment. However, when hEGCs were treated with 50, 100, and 200 pg/mL TNF-α, the embryo implantation rates decreased gradually and significantly (ANOVA p < 0.001, Fig. 6A,C).

### Cu-IUD effects on the endometrium

**Figure 7:**
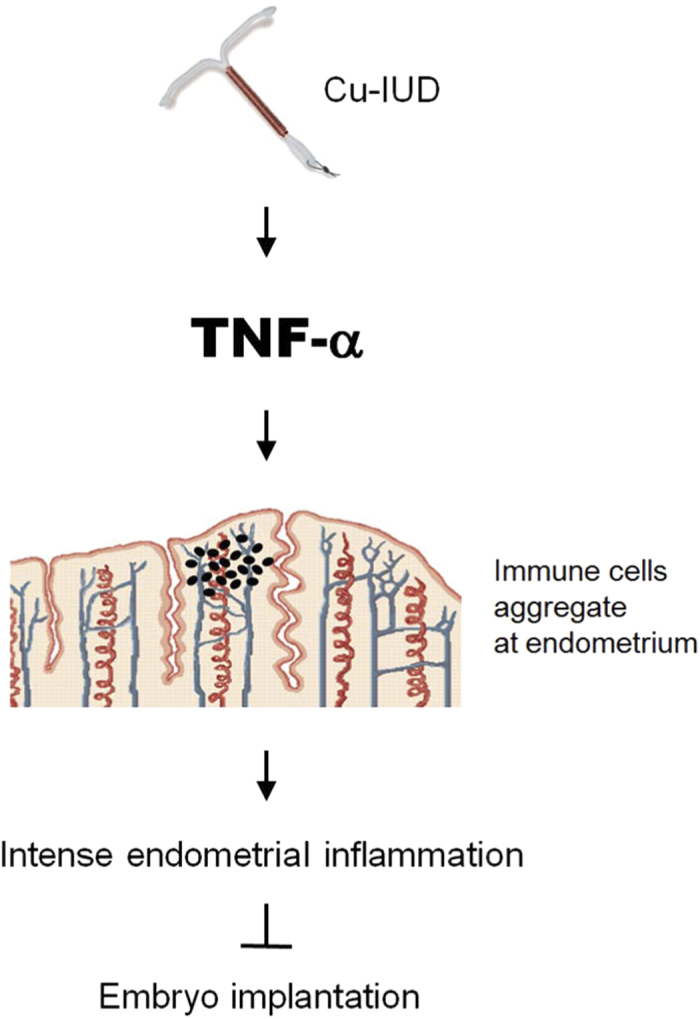
Schematic effects of Cu-IUD reveals that it stimulates endometrial cells to secrete TNF-α. The increased TNF-α concentration results in immune cells aggregation in the endometrium and provokes intense endometrial inflammation, which accordingly interferes with embryo implantation.

Taken together, Cu-IUD induces TNF-α production in hEGCs, which results in immune cells aggregation in the endometrium. Intense endometrial inflammation occurs thereafter, and embryo implantation is prohibited (Fig. 7).

## Discussion

Endometrial inflammation occurs both at peri-implantation period (Group 2) and after the stimulation by Cu-IUD (Group 3), though they have contradictory effects on embryo implantation. To our knowledge, this is the first study comparing the inflammatory cytokine profiles between them. The endometrial inflammation was confirmed by H&E stain, in which subepithelial lymphocytic infiltration was found in both Group 2 and Group 3, but not evident in the control group (Group 1). The endometrial inflammation is more prominent after Cu-IUD stimulation than that occurring at peri-implantation period, in which cytokine array reveals nine cytokines highly expressed in Group 3, whereas only two cytokines are strongly expressed in Group 2.

Cytokines are cell signaling molecules that aid cell to cell communication in immune responses and stimulate the movement of cells towards sites of inflammation, infection and trauma. Cytokine array is employed in this study to investigate the expression profiles of inflammatory molecules. Using the cutoff value of 1.8, two cytokines (IL-1α and TARC) are increasingly expressed in Group 2 and Group 3, as compared with those in Group 1. No cytokine is selectively highly expressed at peri-implantation period, whereas seven cytokines (TNF-α, IFN-γ, IL-1β, MIG, MIP-1β, MIP-2, and TREM-1) are more strongly expressed after the stimulation by Cu-IUD than those in the other two groups.

IL-1α is the one with strong expression both at peri-implantation period and after Cu-IUD stimulation. IL-1 system has been shown to play important roles in the development of pre-implantation embryos^14^. Previous experiments used a mouse model and found that IL-1α was expressed in uterus throughout the peri-implantation period^15^. In addition, women who had a detectable serum IL-1α concentration in IVF-ET cycles on the day of hCG administration achieved a higher implantation rate than those without a detectable IL-1α concentration^16^. However, the favourable-for-embryo-implantation effect of IL-1α might be counteracted by other cytokines like TNF-α in Group 3, and this effect diminishes.

After searching in the literature, TNF-α is probably the cytokine that we are looking for. A high TNF-α concentration is found in endometrial secretions at the peri-implantation period in women with recurrent implantation failure^17^, and it has been found to express strongly after Cu-IUD insertion^18^. TNF-α is produced chiefly by activated macrophages^19^,^20^, though it is also produced by many other cells such as CD4 lymphocytes, NK cells, neutrophils, mast cells, eosinophils, and neurons. The production of TNF-α by human endometrial cells has been reported, and its peak release is in the menstrual phase^21^. Agreeing with it, we demonstrated prominent immunohistochemical TNF-α staining in endometrial cells, as was shown in Fig. 4B.

The primary role of TNF-α is to regulate immune cells and to cause tissue inflammation^22^,^23^. A previous study reported a higher mRNA level of TNF-α in endometria exposed to Cu-IUD^18^. Our results support this finding and reveal that TNF-α is produced *in vitro* by Cu-stimulated hEGCs, and there is a dose-response relationship (Fig. 4A). The recruitment of macrophages (monocytes) in the endometrium by TNF-α is confirmed by an *in vitro* chemotaxis assay in this study (Fig. 5A,B) as well as in a previous report^24^. The aggregation of macrophages, together with other immune cells, results in intense endometrial inflammation, as is shown in Fig. 3, and accordingly interferes with embryo implantation that is demonstrated in an *in vitro* embryo implantation assay (Fig. 6).

Although the endometrial inflammation occurs both at peri-implantation period and after the stimulation by Cu-IUD, the PCNA staining is not different among the three groups (Fig. 3). It implies that the endometrial cells preserve their ability in DNA replication and repair^25^ no matter whether there is endometrial inflammation or not, and irrespective of the intensity of endometrial inflammation.

As in any experimental study, this study has its limitation. The cytokine array employed in this study does not include all the inflammatory molecules. Accordingly, we probably did not find all the differentially expressed inflammatory molecules between the endometria at peri-implantation period and after the stimulation by Cu-IUD. In addition, the cutoff ratio of the expression intensity in the cytokine array was arbitrarily set at 1.8. We might therefore miss some other inflammatory molecules that also play important roles in embryo implantation, as well as in the contraceptive effect of Cu-IUD.

In conclusion, endometrial inflammation occurs both at peri-implantation period and after the stimulation by Cu-IUD. However, the expression profiles of endometrial inflammation are different between them, with the latter more intense than the former. TNF-α is the one with selectively strong expression after Cu-IUD stimulation. It might account for the various biological effects in which the peri-implantation endometrium is favourable for embryo implantation, whereas the endometrium stimulated by Cu-IUD prevents from embryo implantation.

## Methods

### Reagents and antibodies

Antibodies to TNF-α (sc-8301), PCNA (sc-56), F4/80 (sc-59171) and β-actin (sc-47778) were obtained from Santa Cruz Biotechnology (Santa Cruz, CA). CuSO_4_ was purchased from Sigma-Aldrich Co. (St. Louis, MO).

### Expression profiles of endometrial inflammation by mouse cytokine array

Tissue lysate samples were first mixed with the biotinylated detection antibody cocktail at room temperature for 1 hour, while the array membrane was blocked with the blocking solution provided by the manufacturer. The membrane was then incubated with the samples overnight at 2–8 °C on a shaker. After washing, horseradish peroxidase-conjugated streptavidin was added to the membrane for 30 minutes at room temperature, and signal development was achieved by addition of the commercial chemiluminescent detection reagents. Digital imaging system (Bio Pioneer Tech Co.) was used to detect the signals, which were further analysed with Image J^®^ programme. The experiment was conducted in duplicate, and a mean value was calculated.

### Immunohistochemistry

The slides were rehydrated in PBS for 15 min and the endogenous peroxidase was inhibited by 3% H_2_O_2_/methanol for 10 min at room temperature. For blocking, 5% non-fat milk/PBS was used for 30 min at room temperature. Slides were incubated with specific antibodies for TNF-α, PCNA and F4/80 for 16 hours at 4 °C. The peroxidase-conjugated secondary antibodies were incubated for 1 hour at room temperature and were developed by immersing slides in 0.06% 3,3′-diaminobenzidine tetrahydrochloride (DAKO), followed by counterstaining with Gill’s Haematoxylin V. For the quantification of F4/80 immunoexpression, the number of immunopositive cells were counted by eye in 10 fields with a ×400 magnification.

### Isolation of human hEGCs

Human endometrium was obtained from 3 women who had endometrial polyps and underwent hysteroscopic polypectomy. All of them were in the follicular phase at pre-menopausal status. Spare endometrial tissue was collected for experimental use. Informed consent was obtained from each woman before surgery, and this study protocol was approved by the institutional review board (IRB) at our hospital. Since hEGCs are the cells that embryos first interact with during the implantation process^26^, they are used in the *in vitro* experiments in this study. hEGCs purification was done according to the method described before^27^. hEGCs obtained from each woman were pooled together for the following experiments, and they were cultured in phenol-red-free DMEM/F-12 containing 10% charcoal-stripped fetal bovine serum.

### Chemotaxis assay

Chemotaxis assay was done with 24-well transwell inserts (Costar^®^, New York). Conditioned medium (100 μL) obtained from hEGCs culture was placed in the lower well of the chamber and THP-1 cells (5 × 10^6^/mL) were loaded in the upper well. The lower and upper wells were separated by a nitrocellulose philtres with an 8 μm pore size. The chamber was incubated for 2 hours at 37 °C in humidified air with 5% CO_2_. The chamber was disassembled and the cells remaining on the upper surface of the philtre were removed by scraping the philtre with a rubber scraper. The philtres were then fixed and stained with a crystal violet solution (1% w/v crystal violet and 10% v/v methanol). Blue spots developed in positions at which cell migration had occurred. Cells migrating across the philtre onto the lower surface were photographed. Quantification of the migration results were determined by the colour intensity of the spots. Samples on philtres were solubilised in DMSO for 30 minutes at room temperature, and the absorbance was determined at 570 nm with a microplate reader.

### *In vitro* embryo implantation assay

Human BeWo choriocarcinoma cells (ATCC:CCL-98) with GFP-luciferase co-expression were used to produce trophoblast spheroids. Cells were cultured in Ham’s F12K nutrient mixture (pH 7.4) supplemented with 15% FCS in an atmosphere of 5% CO_2_ at 37 °C and subcultured every 3 days by trypsinisation. For the preparation of spheroids, single cells were plated onto noncoated 100-mm plastic petri dishes at 2 × 10^5^ cells/mL in full growth medium. Abundant trophoblast spheroids formed spontaneously by cell aggregation after 24 hours of culture (Fig. 6B). Those with a diameter between 70 and 100 μm were picked using 100 μm and 70 μm nylon cell strainer (Becton Dickinson, Franklin Lakes, NJ). They served as a substitute of blastocysts in the following experiment.

*In vitro* implantation assay was done according to previous reports^28^,^29^ with some modifications. Briefly, the labelled spheroids were added to hEGCs monolayers with different concentrations (0, 10, 50, 100, 200 pg/mL) of TNF-α in 96-well at approximately 500 spheroids per well. After coculture for 30 min at 37 °C in a humidified incubator, the culture medium was removed and the spheroids adherent to hEGCs were examined under the florescence microscope. Relative light unit (RLU) of each well before and after coculture was determined using luminometer (Beckman Coulter DTX 880). *In vitro* implantation rates were calculated according to the formula: (RLU after coculture)/(RLU before coculture) × 100%.

### Statistical analysis

In this study, experiments were repeated for at least three times. The data were examined with one-way ANOVA, followed by Tukey test for multiple comparisons. Data were presented as mean ± SE. Significance level was set as *P* < 0.05 by a two-tailed test. Statistical Programme for Social Sciences (SPSS version 12; SPSS Inc) was used for calculation.

## Additional Information

**How to cite this article**: Chou, C.-H. *et al*. Divergent endometrial inflammatory cytokine expression at peri-implantation period and after the stimulation by copper intrauterine device. *Sci. Rep*. **5**, 15157; doi: 10.1038/srep15157 (2015).
